# Cranial reconstruction utilizing polymeric implants in two different designs: finite element investigation

**DOI:** 10.1186/s12891-024-08066-w

**Published:** 2024-11-20

**Authors:** Yomna H. Shash

**Affiliations:** Biomedical Engineering Department, Faculty of Engineering, Helwan University, Cairo, Egypt

**Keywords:** Cranioplasty, Cranial implant, PEEK, Carbon fiber reinforced PEEK

## Abstract

**Introduction:**

Impact loads applied to the human head can result in skull fractures or other injuries that require a craniectomy. The removed portion is replaced with biological or synthetic materials using cranioplasty surgery. Titanium has been the material of choice for cranial implants due to its superior properties and biocompatibility; however, its issues have prompted the search for substitute materials (e.g., polymers). The issues are related to the requirement for surface modification, casting, radiologic incompatibility and potential allergy risks. Recently, polymeric materials have been used in many fields as alternatives to titanium.

**Objective:**

This research aims to conduct a finite element study to evaluate the skull reconstruction process by using PEEK and carbon fiber reinforced PEEK 30 and 60% in the production of cranial implants as alternatives to conventional titanium implants.

**Materials and methods:**

A three-dimensional model of a defective skull was rehabilitated with a custom-made cranial implant. The implants were stimulated using two designs (plate and mesh), and different polymeric materials (PEEK and carbon fiber reinforced PEEK 30 and 60%) as titanium substitutes, under 2000 N impact force.

**Results:**

The results illustrated that plate implants reduced the stresses on the skull and increased the stresses on brain tissues compared to mesh implants. Titanium, CFR-PEEK 30 & 60% implants (whether mesh or flat) were not prone to fracture, unlike mesh PEEK implants. In addition, CFR-PEEK 60% implants produced the lowest values of stress, strain, and total deformation on the skull and brain compared to titanium implants, unlike PEEK implants. By using the titanium plate implant, the peak tensile and compressive stresses on the skull were 24.99 and 25.88 MPa, respectively. These stresses decreased to 21.6 and 24.24 MPa when using CFR-PEEK 60%, increased to 26.07 and 28.99 MPa with CFR-PEEK 30%, and significantly increased to 41.68 and 87.61 MPa with PEEK. When the titanium mesh implant was used, the peak tensile and compressive stresses on the skull were 29.83 and 33.86 MPa. With CFR-PEEK 60%, these stresses decreased to 27.77 and 30.57 MPa, and with CFR-PEEK 30% and PEEK, the stresses increased to 34.04 and 38.43 MPa, and 44.65 and 125.67 MPa, respectively. For the brain, using the titanium plate implant resulted in peak tensile and compressive stresses of 14.9 and 16.6 Pa. These stresses decreased to 13.7 and 15.2 Pa with CFR-PEEK 60%, and increased to 16.3 and 18.1 Pa, and 73.5 and 80 Pa, with CFR-PEEK 30% and PEEK, respectively. With the titanium mesh implant, the peak tensile and compressive stresses were 12.3 and 13.5 Pa. Using CFR-PEEK 60%, these stresses decreased to 11.2 and 12.4 Pa on the brain, and increased with CFR-PEEK 30% and PEEK to 14.1 and 15.5 Pa, and 53.7 and 62 Pa, respectively. Additionally, the contact area between the PEEK implant (whether mesh or plate design) and the left parietal bone of the skull was expected to be damaged due to excessive strains. Importantly, all implants tested did not exceed permissible limits for tensile and compressive stresses and strains on the brain.

**Conclusion:**

It was concluded that carbon fiber-reinforced PEEK implants, with 30% and 60% reinforcements, can be used as alternatives to titanium for cranial reconstruction. The addition of carbon fibers to the PEEK matrix in these percentages enhances the mechanical, chemical, and thermal properties of the implants. Additionally, these composites are characterized by their low weight, biocompatibility, lack of clinical issues, and ease of fabrication. They can also help preserve the skull, protect the brain, and are not susceptible to damage.

**Clinical significance:**

Overcoming the drawbacks of titanium cranial implants and increasing the effectiveness of the cranioplasty process by utilizing PEEK and carbon fiber reinforced PEEK materials in the reconstruction of the damaged portion of skull.

## Introduction

Human heads are frequently subjected to impact in car accidents, falls, and sporting events [1]. These impact situations are the main causes of mechanically generated brain injuries and unintentional fatality [2]. Increased brain fluid due to swelling and inflammation following injury may increase intracranial pressure and cause brain damage. Excessive pressure can result in irreversible brain damage, coma, or even death [2]. Therefore, a craniectomy is carried out to prevent the clinical problems [3]. Craniectomy is a neurosurgical procedure that involves removing a portion of the skull to eliminate the pressure on the underlying brain [3]. In addition, other conditions require craniotomy and surgical repair which are brain cancers, infections., abscesses, cerebral edema (swelling of the brain), and bleeding within the skull [4].

Cranioplasty is the surgical repair of a cranial bone defect resulting from an injury or a craniectomy procedure [5]. Surgical repair is performed to enhance the morphological and functional anatomy of the cranial vault and to improve the appearance of the skull. Additionally, it seeks to protect the brain from neurological disorders, impairments, or changes in the cerebrospinal fluid [5]. Materials used in cranioplasty can be classified as biological (derived from the patient's or donor's body) or synthetic (e.g., ceramics, metals, and polymers) [6].

The success of cranial reconstruction greatly depends on the choice of implant design and material [7, 8]. In addition, a cranial implant with an attractive design improves social performance and offers psychological comfort [9]. In cranioplasty, neurosurgeons use the preserved bone (autologous bone) from the patient, as an implant to lower intracranial pressure (ICP) and save patients with serious head injuries [9]. Even though autologous bone is the preferred option whenever a cranial defect needs to be repaired, big or irregularly shaped defects, infections, or bone resorption frequently make this impractical [10].

In the absence of autologous bone, traditionally generic mesh and plate implants have been used [5, 6]. Patient-customized implants have become the preferred choice of surgeons due to advancements in imaging techniques like computed tomography (CT) and magnetic resonance imaging (MRI), as well as computational tools like computer-aided design (CAD)/computer-aided manufacturing (CAM) and rapid prototyping techniques like additive manufacturing (AM) and 3D printing [11–14].These techniques offer multiple advantages including good definition on the implant contour, high precision of curvature, reduced risk of surgical complications, and improved aesthetic results. Several crucial requirements must be satisfied when selecting cranial implants, including biocompatibility, adequate mechanical properties, low infection rates, minimal heat conductivity, nonmagnetic characteristics, radiolucent ability, long duration, moldability, and affordability [6].

Titanium (grade 5) is the traditional biomaterial used for bone fixation or reconstruction, and it is thought to have the best biocompatible properties when used for cranioplasty [15]. The advantages of titanium, such as its great mechanical strength, longevity, and chemical and biological compatibility, are the reason for its selection for the fabrication of cranial implants. However, titanium has disadvantages as well, such as the requirement for surface modification, casting problems, and radiologic abnormalities caused by incompatibility with CT and MRI imaging equipment which making it difficult to track the healing process [16, 17]. In addition, titanium implants have a propensity to corrode and deteriorate, which may be harmful because they introduce particles and ions into the surrounding tissues [16]. Moreover, hypersensitivity reactions like erythema, urticaria, eczema, edema, discomfort, and necrosis may be brought on by using the titanium implants [17]. They have also been linked to several clinical issues, including contamination, surface deterioration associated with peri-implantitis and skin defects [18]. Skin defects with mesh titanium implants have been reported in 17.0% of patients [19]. These complications have encouraged the use of other materials, such as ceramics and polymers, as alternatives to titanium.

Recently, the use of polymeric materials in bone reconstruction has attracted much attention [18]. In bone reconstruction, the most prevalent polymeric biomaterials for fabricating custom-made implants are polymethyl methacrylate (PMMA), polyether-ether-ketone ketone (PEKK), polytetrafluoroethylene (PTFE), a polypropylene (PP) and polyether-ether-ketone (PEEK) [20, 21]. Among these polymeric biomaterials, the new high-performance polymeric material PEEK (polyether ether ketone) has a major role in most areas orthopedics due to its biocompatibility and good mechanical, chemical, thermal, and electrical properties [22].

PEEK polymer has high thermal stability inside the human body (melting point 334 °C–343 °C), high toughness and rigidity, excellent fatigue resistance, creep resistance, and excellent sterilization performance [22]. In addition, it is non-toxic and does not have any harmful reactions or release any harmful constituents. PEEK and its composites also have natural radiolucency, X-ray, CT, ultrasonic, and MRI compatibility, making follow-up procedures easier. In cranioplasty, PEEK is superior to titanium in both cosmetic satisfaction outcomes and brain function improvement, according to [22]. The main advantages of PEEK material are its low modulus of elasticity and high shock-absorbing ability; thus, it is expected to dampen the stresses generated on the skull and underlying brain tissues. However, more research is needed to confirm the possibility of using this soft material instead of titanium in the reconstruction of a damaged skull, and detect its effects on the surrounding bone structure and underlying brain tissues [23].The osseointegration potential of PEEK has been improved by surface modification through chemical or physical methods, incorporating bioactive materials like hydroxyapatite as composites or surface coatings; or creating three-dimensionally porous structures on its surfaces [24].

The stiffness of PEEK can be improved to meet biological demands by reinforcing carbon fibers with diameters ranging from a few microns to ten microns [22, 23]. PEEK has an elastic modulus of approximately 3.5 GPa; by reinforcing it with 30% carbon fibers, the elastic modulus increases to 18 GPa, representing semi-stiff material with an elastic modulus close to the bone [24]. Carbon fibre-reinforced PEEK 60% represents the upper limit of the elastic modulus (150 GPa) of PEEK compounds, which is higher than that of titanium (110 GPa), according to the manufacturer (Invibio Ltd., Thornton-Cleveleys, UK) [24, 25]. These PEEK composites are characterized by their strengths, biocompatibility, resistance to chemical erosion, compatibility with imaging techniques (no artifacts appear in images), low plaque affinity, and high inertness, with no clinical issues [23, 24]. In addition, they are less expensive than metallic and ceramic materials and easier to manufacture in different shapes and sizes.

The finite element method (FEM) has gained widespread recognition among medical professionals and researchers as a means of solving biomechanical issues. FEM has emerged as a useful tool in the field of cranial construction for evaluating cranial implant designs and materials [7, 8]. It is capable of precisely reconstructing intricate geometries, altering flaws, suggesting alternative designs, stimulating various materials in various scenarios, and extracting internal stresses and strains at any point [8].

Several tests have been conducted to determine the properties of polymeric materials under different conditions [26–28]. However, few in vivo and in vitro studies were carried out on polymeric cranial implants and evaluated their effects on the surrounding bone and underlying tissues [29, 30]. Consequently, further research is necessary to evaluate the biomechanical performance of polymeric cranial implants in terms of mechanical resistance, their effects on the skull and brain, and the performance of the reconstruction process. Therefore, this research aims to investigate the possibility of using PEEK and its composites (carbon fiber reinforced PEEK) with different stiffness and designs in the production of cranial implants as alternatives to the traditional titanium implants, and evaluate their mechanical performance to enhance the skull reconstruction process using the finite element method (FEM). Stress–strain analyses are conducted on the defective skull rehabilitated with a cranial implant, using two designs (plate and mesh) and innovative PEEK and PEEK composites as alternatives to titanium, under impact loading scenario. The stimulated materials for the cranial implants are traditional titanium, soft PEEK, stiff PEEK composite (CFR-PEEK 60%), and semi-stiff PEEK composite (CFR-PEEK 30%). To evaluate the results, the maximum von Mises stresses, the total deformations, and the peak tensile and compressive stresses are extracted and analyzed.

## Materials & methods

### Model generation

Based on computerized tomography (CT) images, a complicated intact (healthy) model of the skull, including the brain, was downloaded as OBJ files from the anatomical database "BodyParts3D/Anatomography (BodyParts3D, Life Sciences Integrated Database Center, Japan) [31]". A 22-year-old volunteer with a body mass index of 21.7 had CT scans taken every 2 mm from the top of the head to the feet for this database [32]. In image acquisition, the volunteer was lying down on his back, with his hands resting on his sides and his feet and ankles positioned to suggest that he was standing.

**Fig. 1:**
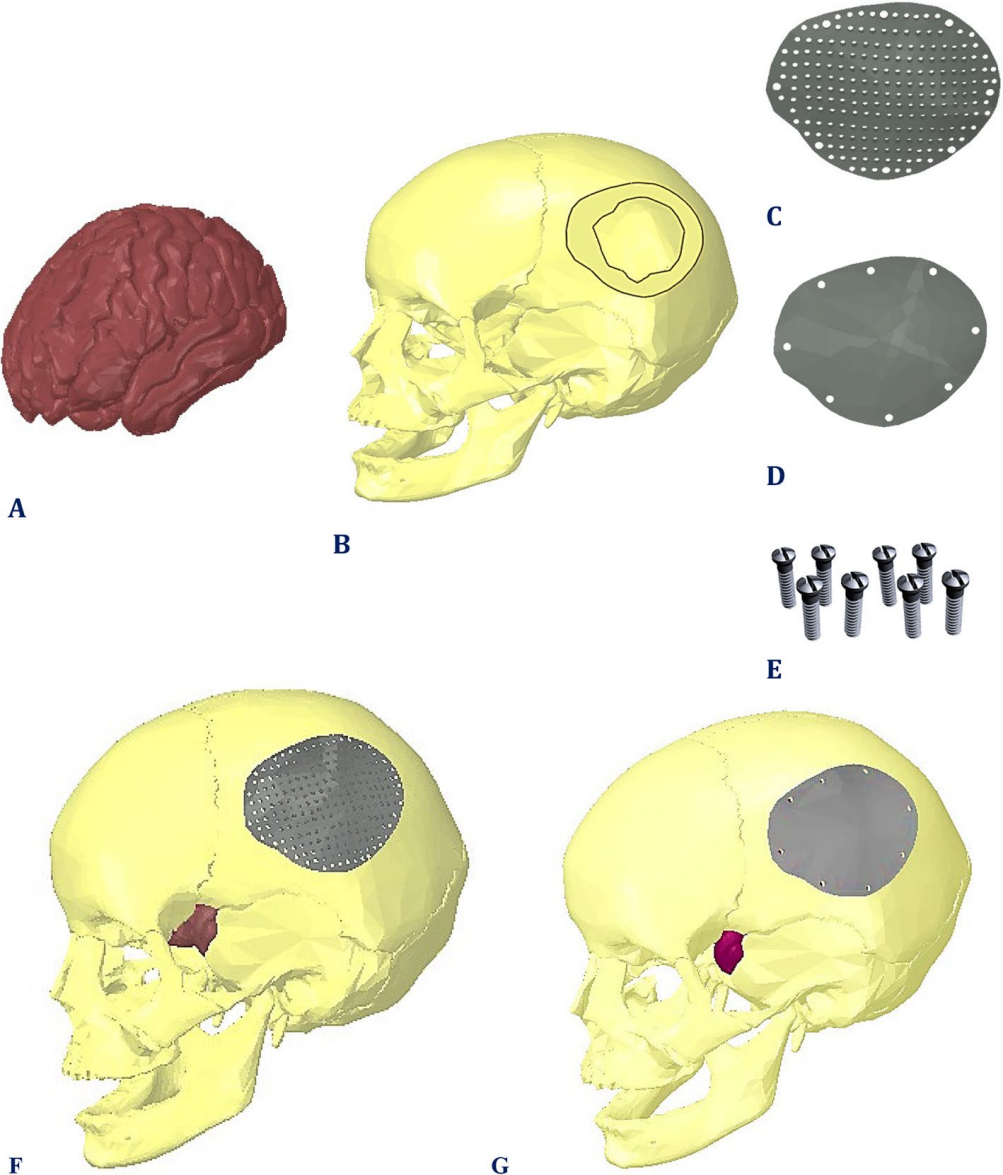
Model parts: **A**) brain, **B**) defective skull, **C**) mesh implant, **D**) plate implant, **E**) eight fixation mini-screws, **F**) Final model with mesh implant, and **G**) Final model with plate implant

The skull and brain (Fig. 1A) models were transformed into solid, altered, and repaired with the aid of the software "Space Claim". The complex geometries were fixed by using the repair tools to fix the curves, gaps, edges, bad faces, and missing faces. The repair process also included merging faces, removing small faces, and simplifying complex faces and curves.

A large defect was constructed in the left parietal bone to mimic an unhealthy case (Fig. 1B). To design the cranial implant, the patient's skull was assumed to be symmetrical along its mid-sagittal plane. Two different designs for implants were constructed with irregular shapes to suit the defective portion of the skull as shown in Fig. 1. The first design was a plate implant with no holes, while the other design was a mesh implant with many holes with diameters of 2 mm and interspaces of 2 mm, as shown in Fig. 1C and D, according to [30, 33, 34]. Each implant had a thickness ranging from 2 mm at the peripheral to 4 mm at the center. Eight fixation points with 1.5 mm radius mini-screws (Fig. 1E) were used for the plate and mesh implants to ensure tight and stable implant fixation within the defective skull. After that, all components (skull, implants, screws and brain) were assembled and exported to ANSYS software (ANSYS 18.1, Houston, TX, USA). Figure 1F and G illustrate the final models after cranioplasty process.

**Fig. 2:**
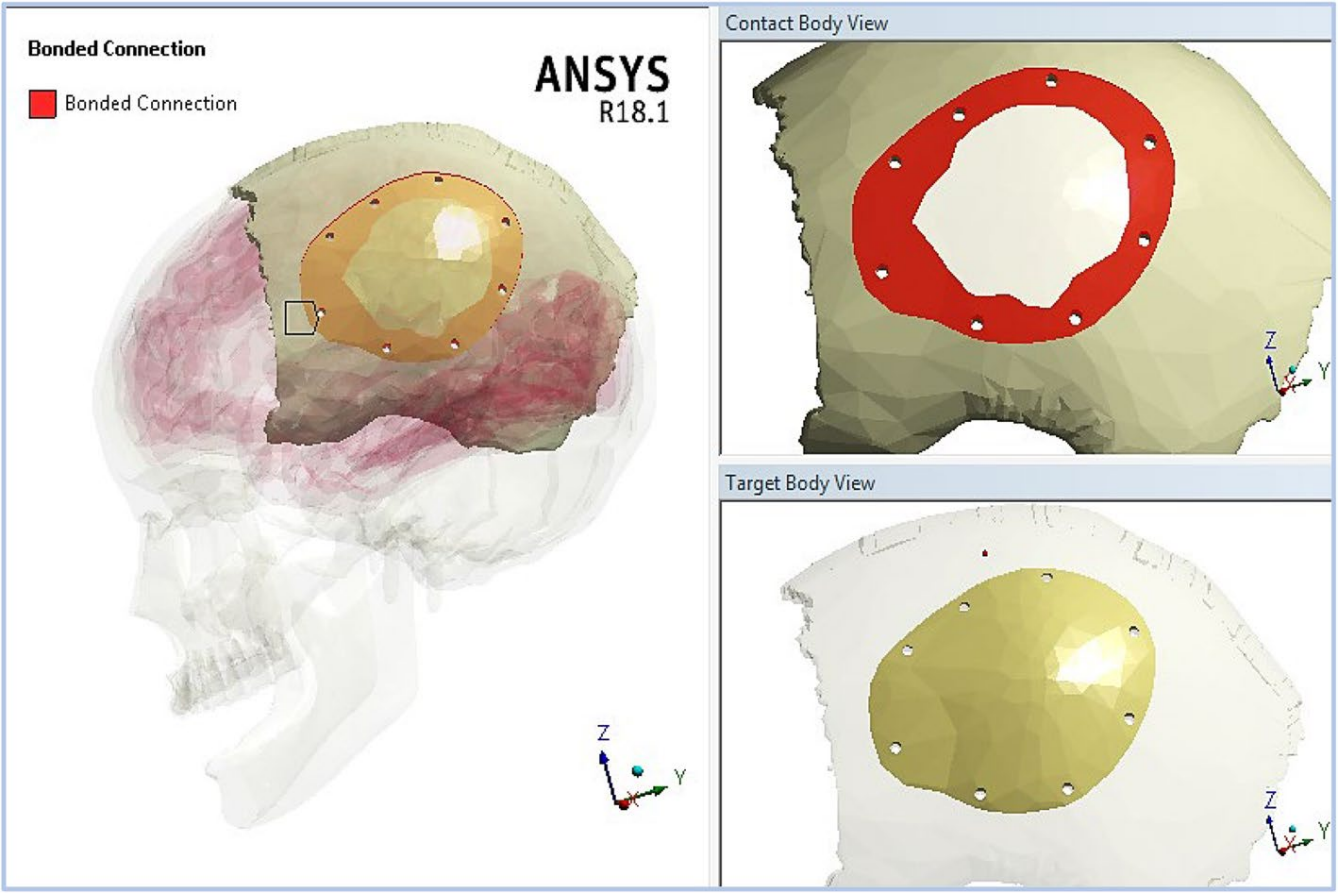
Bonded connection between left parietal bone and implant

In Ansys software, the connection between the model parts were adjusted. The screws were in bonded connection with the implants and skull. In addition, delayed (rather than immediate) loading condition was assumed in this study, involving a waiting period after surgery to ensure complete osseointegration between the implant and the skull (Fig. 2). Besides, a sliding connection were set between the skull and brain due to the presence of cerebro-spinal fluid (CSF).

### Materials selection

In this paper, cranial implants with two designs were simulated using different materials, including the traditional material (titanium), soft polymer (PEEK), semi stiff polymeric composite (carbon fiber reinforced PEEK 30%), and stiff polymeric composite (carbon fiber reinforced PEEK 60%). The mechanical properties of these materials are shown in Table 1 [24, 25, 34].

**Table 1:** The properties of the skull and implants

	**Young's Modulus (MPa)**	**Poisson's Ratio**	**Density (kg/m3)**	**Tensile Yield Strength (MPa)**	**Compressive Yield Strength (MPa)**
Skull	Ex = 20,000 Gxy = 4.53 Ey = 12,000 Gyz = 4.53 Ez = 12,000 Gzx = 4.53	0.235 0.376 0.376	1800	93–100	133–180
Implant					
*• Titanium*	110,000	0.34	4500	880	970
*• CFR-PEEK 60%*	150,000	0.35	1600	2000	800
*• CFR-PEEK 30%*	18,000	0.35	1370	200	300
*• PEEK*	3,500	0.4	1300	120	120

The properties of skull and intracranial contents were taken from the previous studies [35–43], and are shown in Tables 1 and 2. The skull was modeled with orthotropic properties, as the bone is a complex composite and is frequently modeled as orthotropic material rather than isotropic [39]. The brain and cerebro-spinal fluid (CSF) were modeled using the viscoelastic model (Table 2) described with the following equation [40]:

**Table 2:** The viscoelastic properties of the brain and CSF

	**Poisson's Ratio**	**Density (kg/m3)**	**K (MPa)**	**GO (KPa)**	**G (KPa)**	**Β (KPa)**	**Tensile Yield Strength (MPa)**	**Compressive Yield Strength (MPa)**
Brain	0.45	1,040	2,190	6	0.1	80	1e-3	50e-3
CSF	0.3	1,040	2,190	0.5	0.1	80	-	-

Where K, Bulk Modulus; Go, Short Time Shear Modulus; G, Long Time Shear Modulus; and β, Decay Coefficient

$$G\left(t\right)\;=\;G1+\left(G0+G1\right)\;e^{\beta t}$$

Where G1 is long-time shear modulus, G0 is short-time modulus, β is decay coefficient and t is time.

### Mesh adjustment

**Fig. 3:**
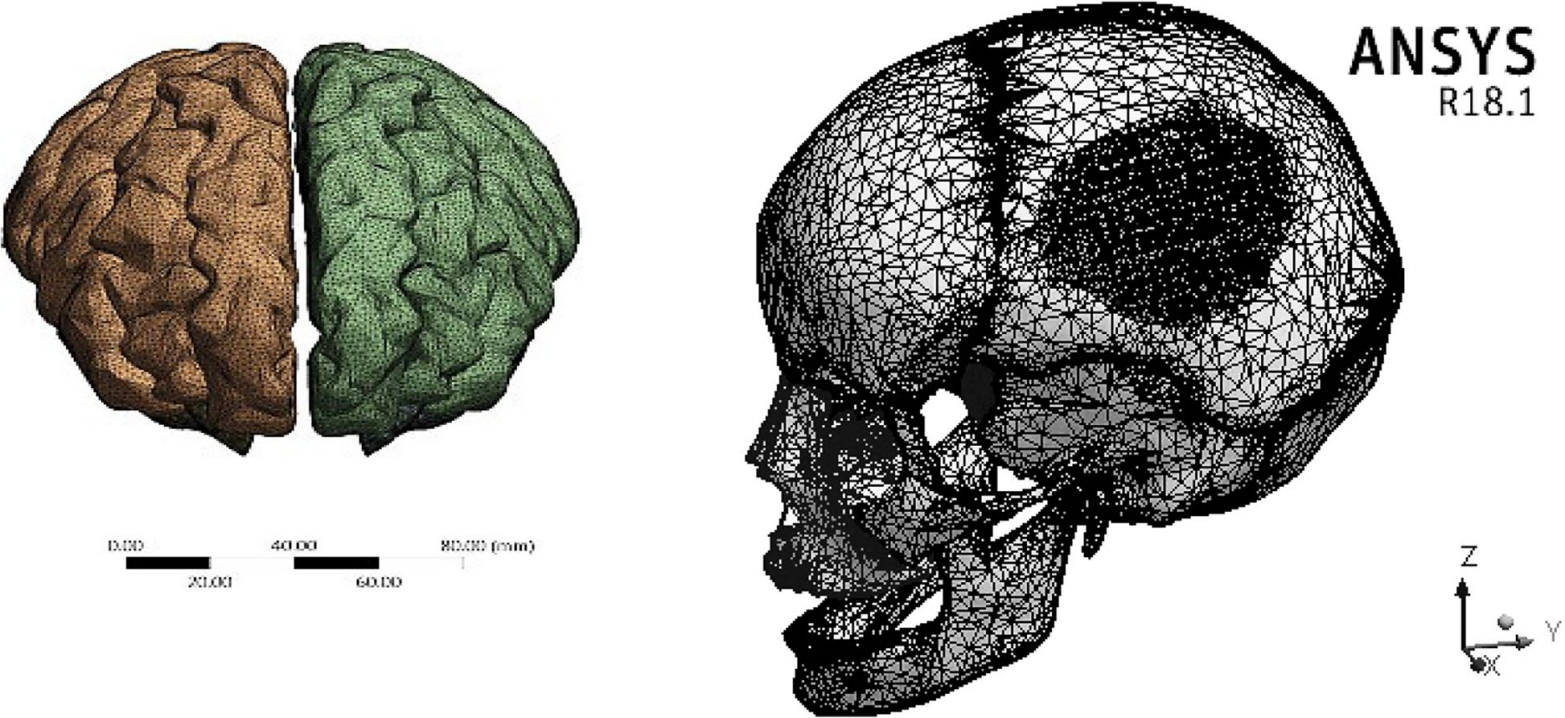
Mesh setting

**Fig. 4:**
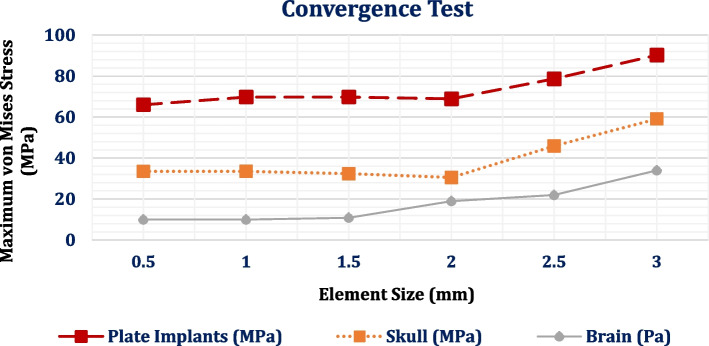
Convergence Test: the relation between element size (mm) and the maximum von Mises stresses on plate implants, skull and brain

One important factor in improving the accuracy of the finite element method findings is the mesh division method [44]. The ANSYS program produced a huge three-dimensional mesh of elements and nodes by using the "Adaptive" size function with "tetrahedral" mesh and "0.5–1.5 mm" element size (Fig. 3 and Table 3). The span angle was adjusted to fine and the smoothing factor was set high to can cover every section of the model. The mesh refinement was established based on the convergence test. Figure 4 shows the relationship between the element size (0.5–3 mm) and the extracted von Mises stresses on the plate titanium implant, skull, and brain.

**Table 3:** The number of elements and nodes

	**No. of Elements**	**No. of Nodes**
Brain	706287	11119773
Skull	787024	1352476
Mesh Implant	29964	56722
Plate Implant	22788	53943

### Static structural analysis

**Fig. 5:**
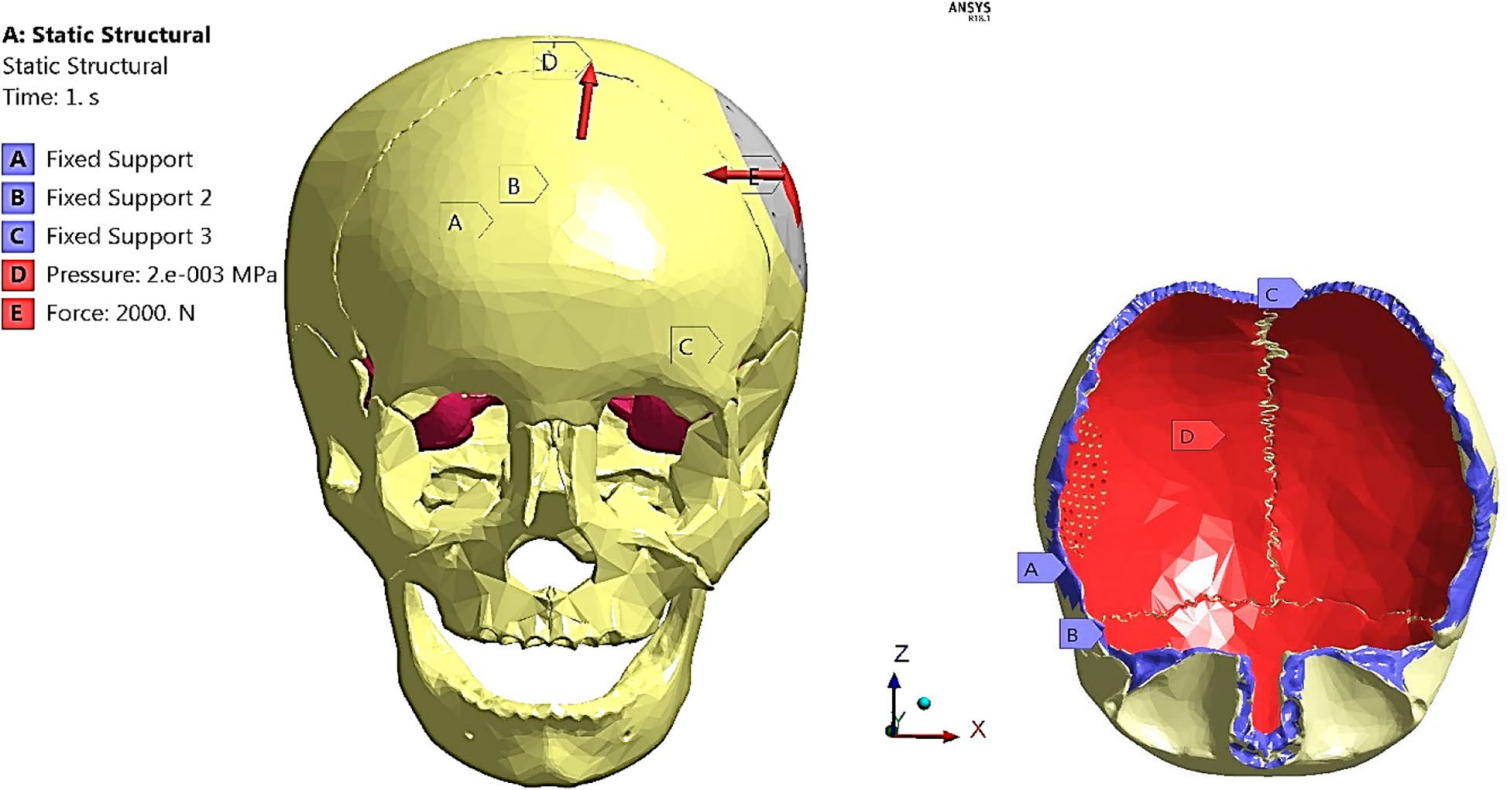
Boundary conditions

Three distinct boundary conditions—external force, intracranial pressure, and fixed support—were established in order to assess the performance of implant design and material. According to [8, 30, 45], an external force of nearly 2000 N was applied on the skull during real-world scenarios related to collision forces, trauma cases, free falls, and road accidents. Consequently, in the present study, a uniformly distributed static load of 2000 N was applied perpendicularly to the plate and mesh implants in the negative direction of the x-axis, over an area of approximately of 3 mm^2^ as shown in Fig. 5.

Intracranial pressure (ICP) is the term for the internal pressure that exists naturally within the skull. The pressure varies with age, and body position and affects the cranial implant after surgery [30, 45]. For an adult, the normal range of ICP is 7–15 mm Hg (~ 900- 2000 Pascal). In this study, to replicate the intracranial pressure conditions, a static pressure of 2000 Pascal (2 e-003 MPa) was applied to the inner surface of the skull and was uniformly distributed over the implant area and the brain [46]. Concerning the boundary conditions, the nodes of the inferior border of the parietal bones were constrained in all directions to prevent movement of the model under the loading effect, as shown in Fig. 5.

### Results extraction and evaluation

Analyses were carried out using ANSYS software to extract the von Mises stresses, the total deformations and the tensile and compressive stresses and strains. The von Mises stresses were extracted to find the relations between implant stiffness and the generated maximum stresses on implants, skull, and brain, while the total deformations were important to determine the changes in the shape or size due to the applied load.

For evaluating the endurances of implants with two designs and different materials, the maximum tensile stresses (peak maximum principal stresses) and maximum compressive stresses (peak negative minimum principal stresses) were extracted and then compared to the tensile and compressive yield limits, according to the principal stress theory [47, 48], and earlier research [30, 49, 50].

For biological parts (skull and brain), the maximum tensile and compressive stresses were extracted to evaluate their responses by comparing them with the tensile and compressive yield limits, respectively (shown in Table 1) [47, 48]. Additionally, the estimated tensile and compressive strains were compared to the allowable limits because of the potential for tissue microdamage due to the concentration of excessive strains. The skull is damaged when the strain exceeds the thresholds of 5000–6000 με in compression and 2500–3000 με in tension [49]. For the brain, the strain level exceeding 0.1 (100,000 με), 0.2 (200,000 με), and 0.25 (250,000 με) causes reversible damage, functional damage, and structural damage respectively, in tension and compression [34].

## Results

### Validation study

Firstly, in this paper, the intact model of the skull was validated with Pajic et al*.* study [40]. The intact model was constructed using computed tomography scans of a human head with frontal sinus cavities that were normally formed. A force of 7.7 KN was applied perpendicularly to the forehead over a circular area measuring 2 cm in diameter to stimulate a frontal hit. The bone and teeth were modeled as linear elastic materials, defined with Young’s modulus, Poisson’s ratio, and density, while the brain was modeled using the viscoelastic model, to mimic Pajic et al*.* study [40].

**Fig. 6:**
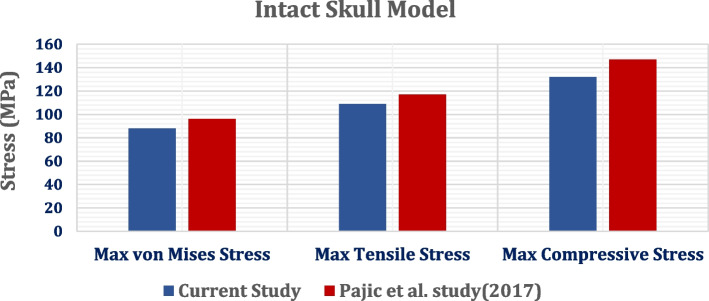
Comparison between the current model and Pajic et al. model [40]

To estimate the risk of skull fracture, the maximum tensile and compressive stresses were extracted and compared with the limits of 92 MPa in tension and 133 MPa in compression, according to Pajic et al*.* study [40]. The FEA models were discretized into the "fine" volume mesh, using the linear tetrahedron elements available in the ANSYS Meshing module. The distribution of von Mises stress, maximum, and minimum principal stresses were analyzed (Fig. 6). The extracted maximum von Mises stress, and maximum tensile and compressive stresses of the current study were less than the stresses extracted by Pajic et al*.* [40]. However, the differences between the results did not exceed 10%.

Another validation study was carried out with Godinho et al*.* [50] study. This study provided a further understanding of the reliability of the finite element analysis by presenting a validation study that compared the deformations of a finite element model of a real cadaveric human skull to those of the same model under equivalent simulated loading. To mimic Godinho et al*.* [50] study, the skull was extremely well preserved, with no missing skeletal elements. Then the head was segmented, stimulated with the same mechanical properties, and mechanically loaded with a compressive vertical force of 750 N which applied to the frontal bone. In Godinho et al. [50] study, the maximum (ε1) and minimum (ε3) principal strains were calculated and the result illustrated that the tensile strains on the loaded area had a range of 300–1600 με, while the compressive strains had a range of 600-2400με. In the current study, the tensile and compressive strains had approximate ranges of 210–1439 με and 510 – 2242 με, respectively.

### Stresses on implant

**Fig. 7:**
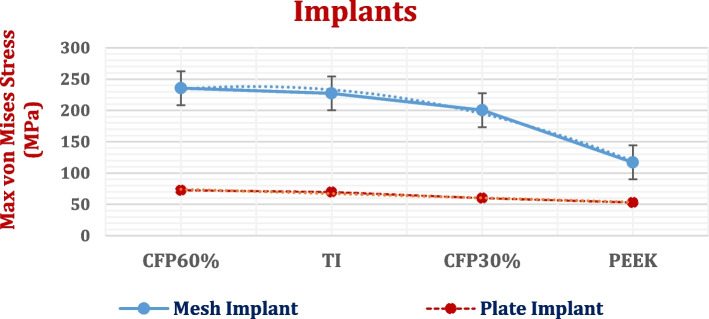
Maximum von Mises stresses (MPa) on mesh and plate implants with different materials. (*P*-Value < 0.05)

The maximum von Mises stresses (a measure of the intensity of the multiaxial stress state) on plate and mesh implants were extracted in Fig. 7 with standard error. The *p*-value was calculated for mesh implants, in comparison to the plate implants. As shown in Fig. 7, the maximum von Mises stresses on the mesh implants were higher than those on the plate implants, due to the presence of details and holes, resulting in a *p*-value of lower than 0.05 representing the statistically significant.

Compared with the mesh titanium implant, the mesh CFR-PEEK 60% implant increased the stress by 3.6%. Conversely, the stresses on the mesh CFR-PEEK 30% and PEEK implants were reduced by 11.79% and 48.53%, respectively. Figure 7 also shows that in comparison to the titanium plate implant, the stress on plate CFR-PEEK 60% implant increased by 3.98%; however, the stresses on plate CFR-PEEK 30% and PEEK implants decreased by 14% and 24%, respectively.

In addition, for evaluating the endurances or fracture risk of polymeric implants with two designs, the maximum tensile stresses (peak maximum principal stresses) and maximum compressive stresses (peak negative minimum principal stresses) were extracted as illustrated in Table 3 and compared to the tensile and compressive limits (Table 1), according to principal stress failure criteria [47, 48]. The *p*-value was calculated for each material compared to titanium.

Compared with that of the mesh titanium implant, the peak tensile stress of the mesh CFR-PEEK 60% implant nearly did not change; However, the peak compressive stress increased by 2%. The peak tensile and compressive stresses of the mesh CFR-PEEK 30% implant decreased by 14.17% and 12.51%, respectively, compared to titanium implant. With the use of mesh PEEK implant, the peak tensile and compressive stresses decreased by 23.24% and 45.68%, respectively.

For the plate implants, in comparison to titanium, the peak tensile and compressive stresses increased by (3.96% and 2.03%) on CFR-PEEK 60% implant; however, decreased by (34.73% and 9.91%) and (44.8% and 21.64%) on CFR-PEEK 30% and PEEK implants, respectively.

**Fig. 8:**
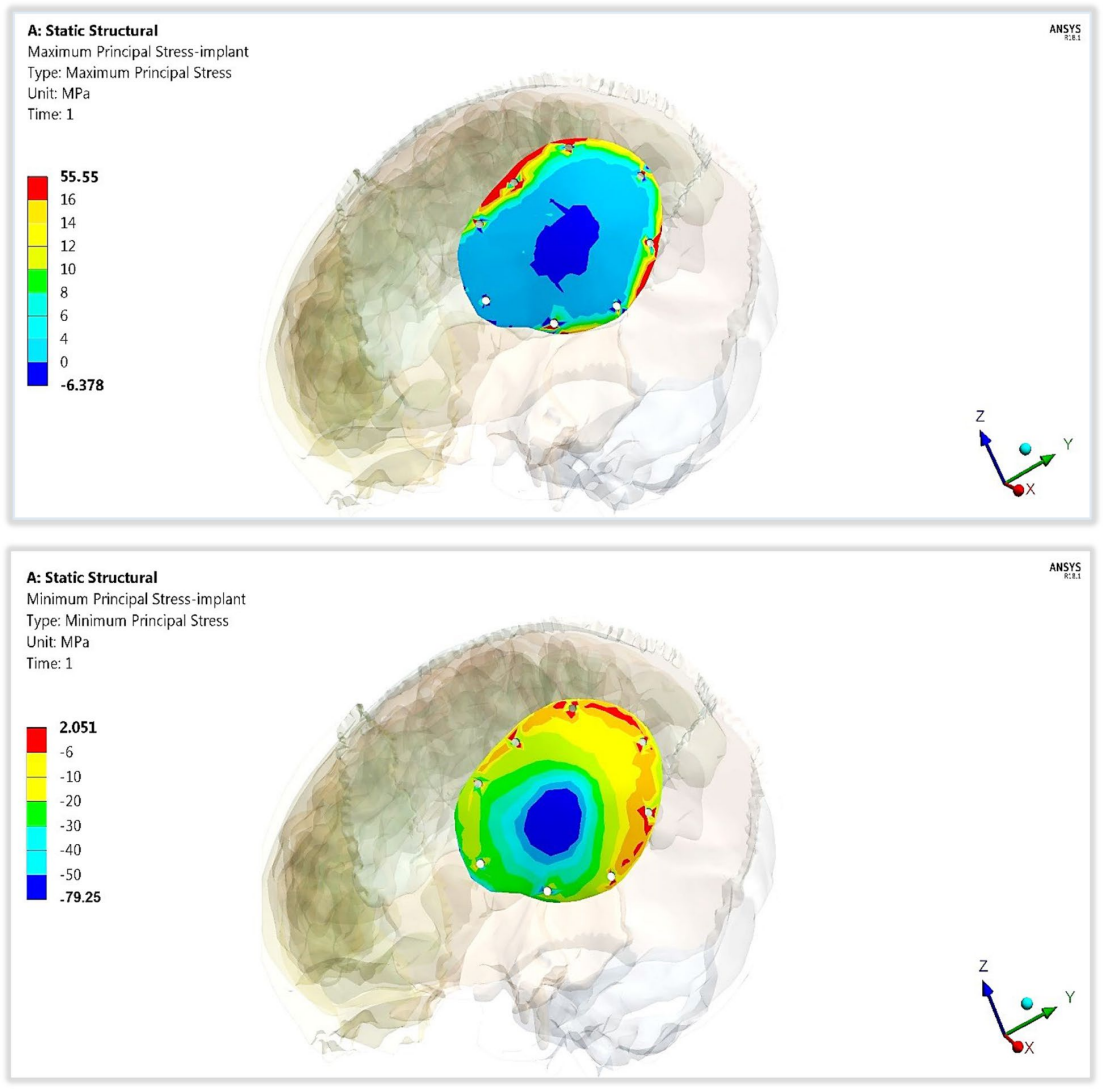
The distributions of maximum and minimum principal stresses (MPa) on plate CFR-PEEK 60% implant

**Fig. 9:**
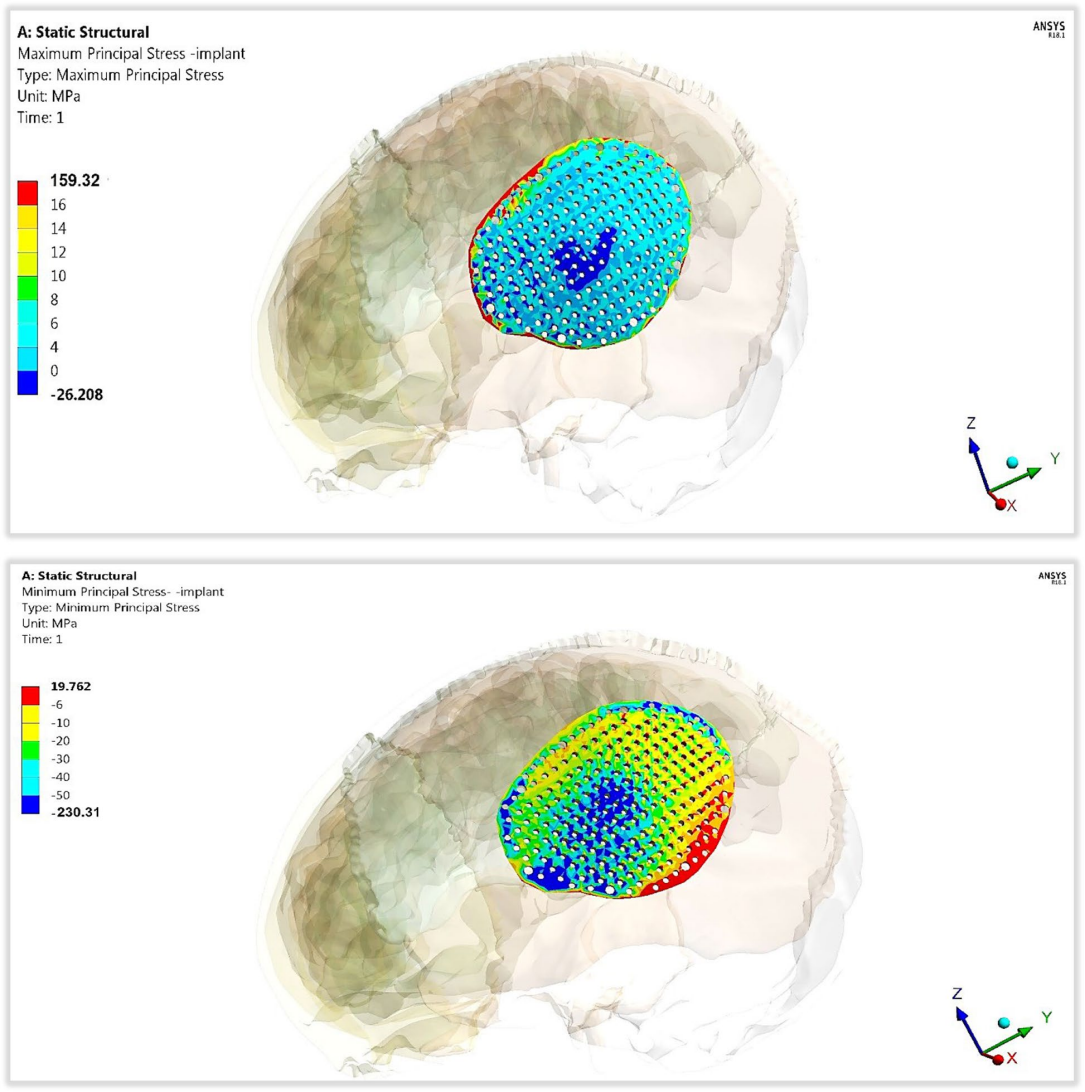
The distributions of maximum and minimum principal stresses (MPa) on mesh CFR-PEEK 60% implant

Figures 8 and 9 illustrate the distributions of maximum and minimum principal stresses on the plate and mesh CFR-PEEK 60% implants under a 2000 N load. As shown in figures, the tensile and compressive stresses had nearly uniform patterns on the plate implant, in contrast to the mesh implant.

### Stresses and strains on skull

**Fig. 10:**
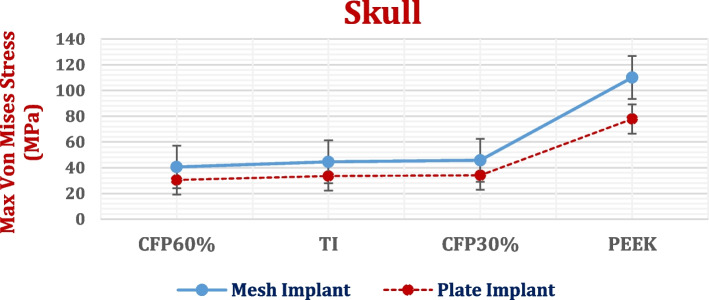
Maximum von Mises stress (MPa) on the left parietal bone of skull using different implant designs and materials. (*P*-value < 0.05)

**Fig. 11:**
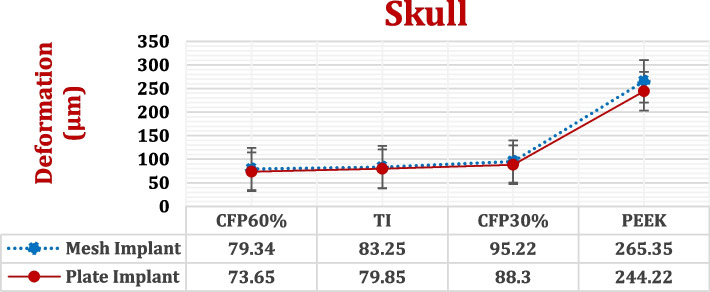
Total deformation (μm) of the left parietal bone of skull using different implant designs and materials. (*P*-value > 0.05)

**Fig. 12:**
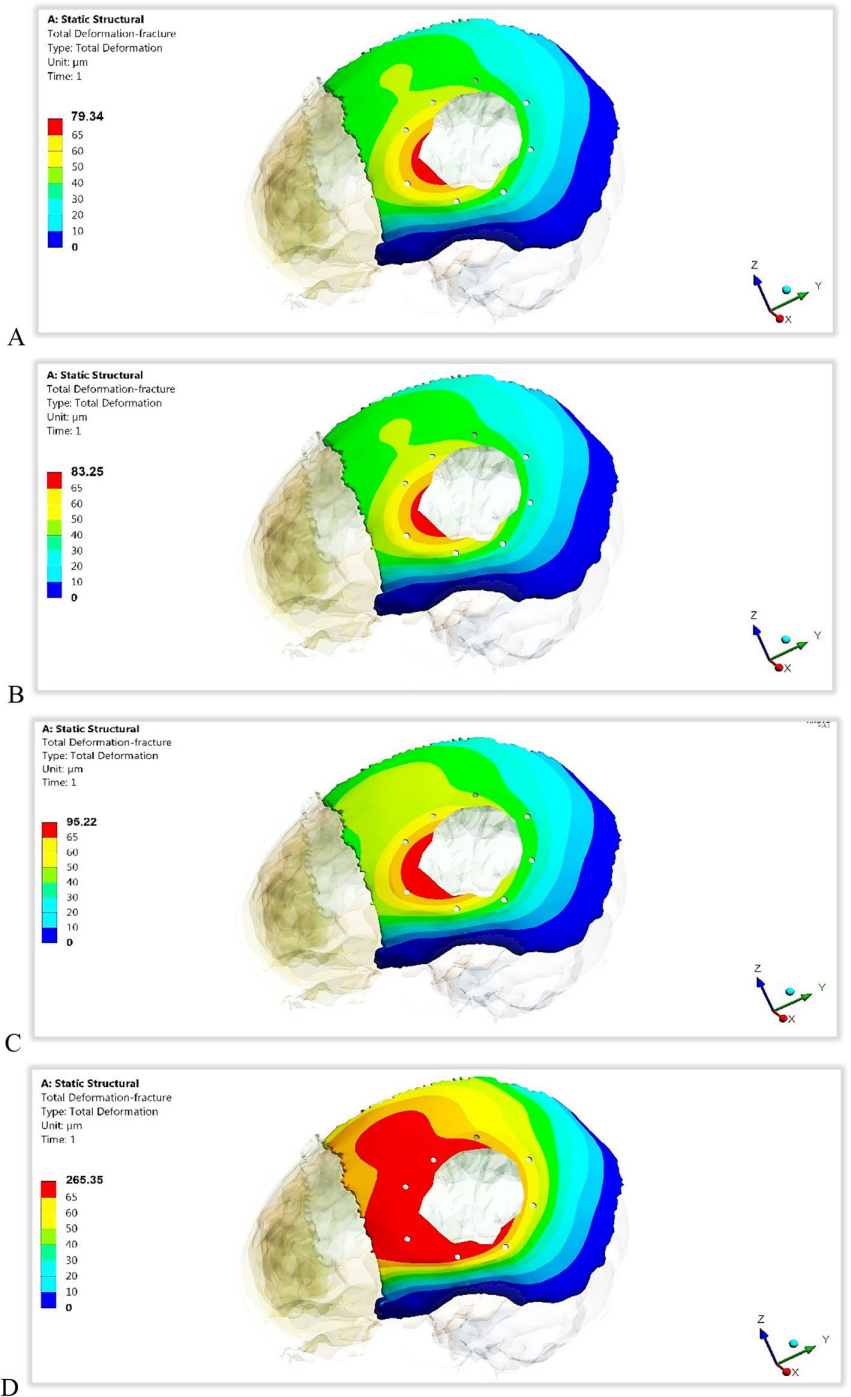
The distribution of total deformations (μm) on the left parietal bone of skull using different mesh implant materials: A) CFR-PEEK 60%, B) Titanium, C) CFR-PEEK 30% and D) PEEK

For the left parietal bone of skull, the maximum von Mises stresses, deformations, and peak tensile and compressive stresses and strains were extracted by using both mesh and plate implants with PEEK and CFR-PEEK 30 and 60% materials, as shown in Figs. 10 and 11 and Tables 4 and 5 with calculating the *p*-values. The von Mises stresses were extracted to find the relations between implant stiffness and the generated stress on skull. As illustrated in Fig. 11, both plate and mesh implants produced nearly the same deformation on the skull for all the materials used, resulting in *p*-value higher than 0.05. Figure 12 illustrates the distribution of total deformations on the skull using mesh implants. As shown in Fig. 12, the soft PEEK implants produced the highest deformations on the skull, compared to the semi-stiff CFR-PEEK 60% implant and the stiff titanium and CFR-PEEK 60% implants.

**Table 4:** Peak tensile and compressive stresses (MPa) on the left parietal bone of skull using different implant designs and materials

		***TI***	***CFR-PEEK60%***	***CFR-PEEK30%***	***PEEK***
**Mesh Implant**	***Tensile***	29.83	27.77	34.04	44.65
	***Compressive***	33.86	30.57	38.43	125.67
**Plate Implant**	***Tensile***	24.99	21.64	26.07	41.68
	***Compressive***	25.88	24.24	28.99	87.61
***P*-Value**			**0.004**	**0.012**	**0.01**

**Table 5:** Peak tensile and compressive strains (με) on the left parietal bone of skull using different implant designs and materials

		***TI***	***CFR-PEEK60%***	***CFR-PEEK30%***	***PEEK***
**Mesh Implant**	***Tensile***	1998	1922	2169	4123
	***Compressive***	2169	2122	2254	8090
**Plate Implant**	***Tensile***	1901	1813	2127	3907
	***Compressive***	2011	1945	2074	6730
***P*-Value**			**0.002**	**0.018**	**0.015**

With the use of a mesh CFR-PEEK 60% implant, the maximum von Mises stress reduced by 8.9% compared to the titanium mesh implant; besides, the total deformation reduced by 4.69% compared to that of the titanium implant. The peak tensile and compressive stresses reduced by 6.91% and 9.72% respectively, and the peak tensile and compressive strains reduced by 3.8% and 2.16%. With the use of a mesh CFR-PEEK 30% implant, the maximum von Mises stress increased by 2.78%, and the total deformation increased by 14.37% compared to that with the use of a titanium implant. The peak tensile and compressive stresses also increased by 14.11% and 13.49%, respectively, and the peak tensile and compressive strains increased by 8.56% and 3.92%, respectively. By using a mesh PEEK implant, the maximum von Mises stresses, deformations, and tensile and compressive stresses and strains greatly increased, compared to a titanium mesh implant.

By using plate implant, maximum von Mises stress and the total deformation on skull using a plate CFR-PEEK 60% implant reduced by 9.10% and 7.76%, compared to titanium implant. In addition, the peak tensile and compressive stresses reduced by 13.4% and 6.34%, respectively, and the peak tensile and compressive strains reduced by 4.62% and 3.28%, respectively. Compared with that of the titanium implant, the maximum von Mises stress on the skull using the plate CFR-PEEK 30% implant increased by 1.7%, and the total deformation increased by 10.58%. Similarly, the peak tensile and compressive stresses increased by 4.32% and 12%, respectively, and the maximum tensile and compressive strains increased by 11.8% and 3.13%, respectively. When using a plate PEEK implant instead of titanium implant, there was a significant increase in the maximum von Mises stresses, deformations, and peak tensile and compressive stresses and strains.

**Fig. 13:**
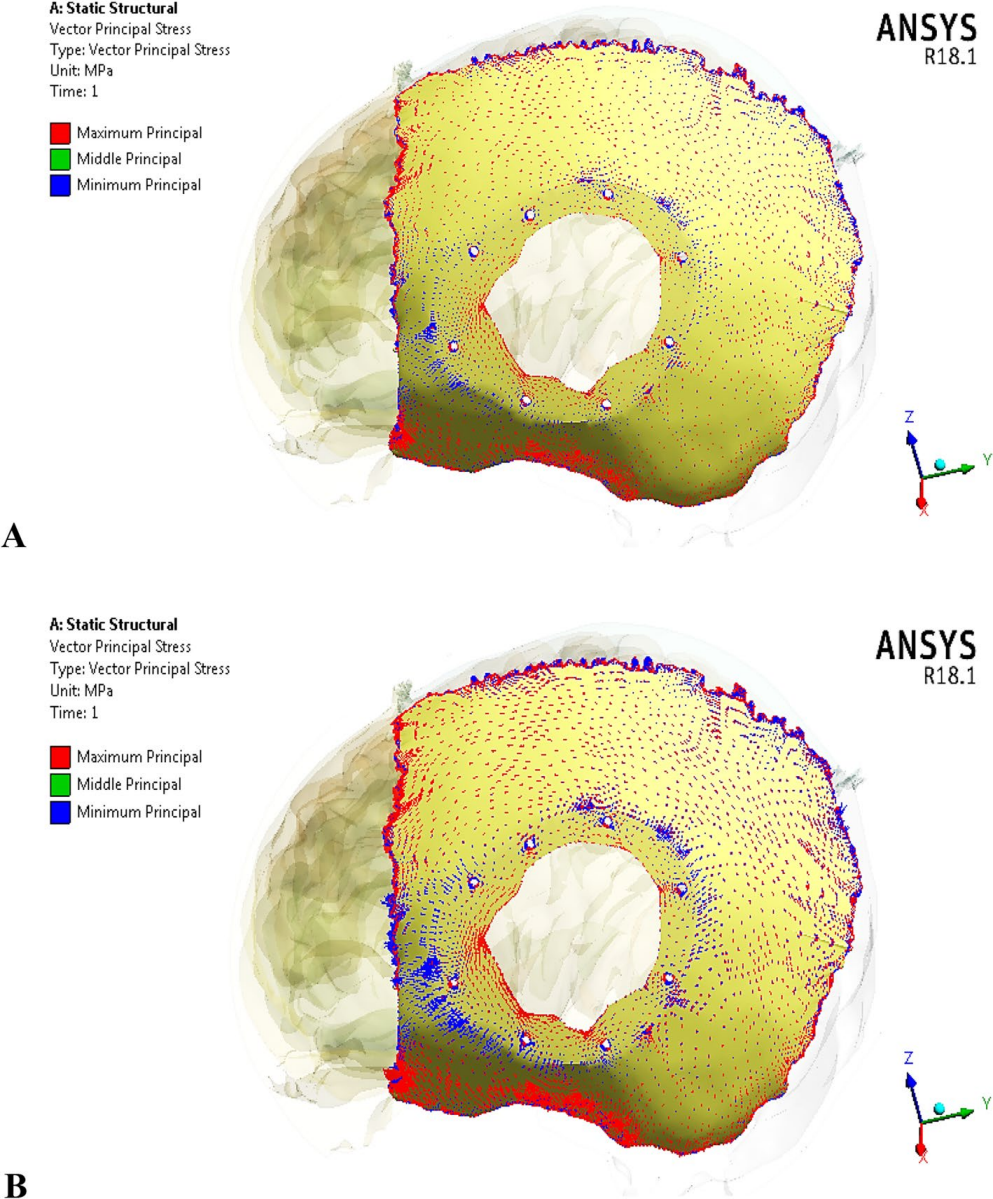
Principal stress vectors for left parietal bone using plate implants: **A** CFR-PEEK 60%, and **B** PEEK. Where red arrows (tension) and blue arrows (compression)

**Fig. 14:**
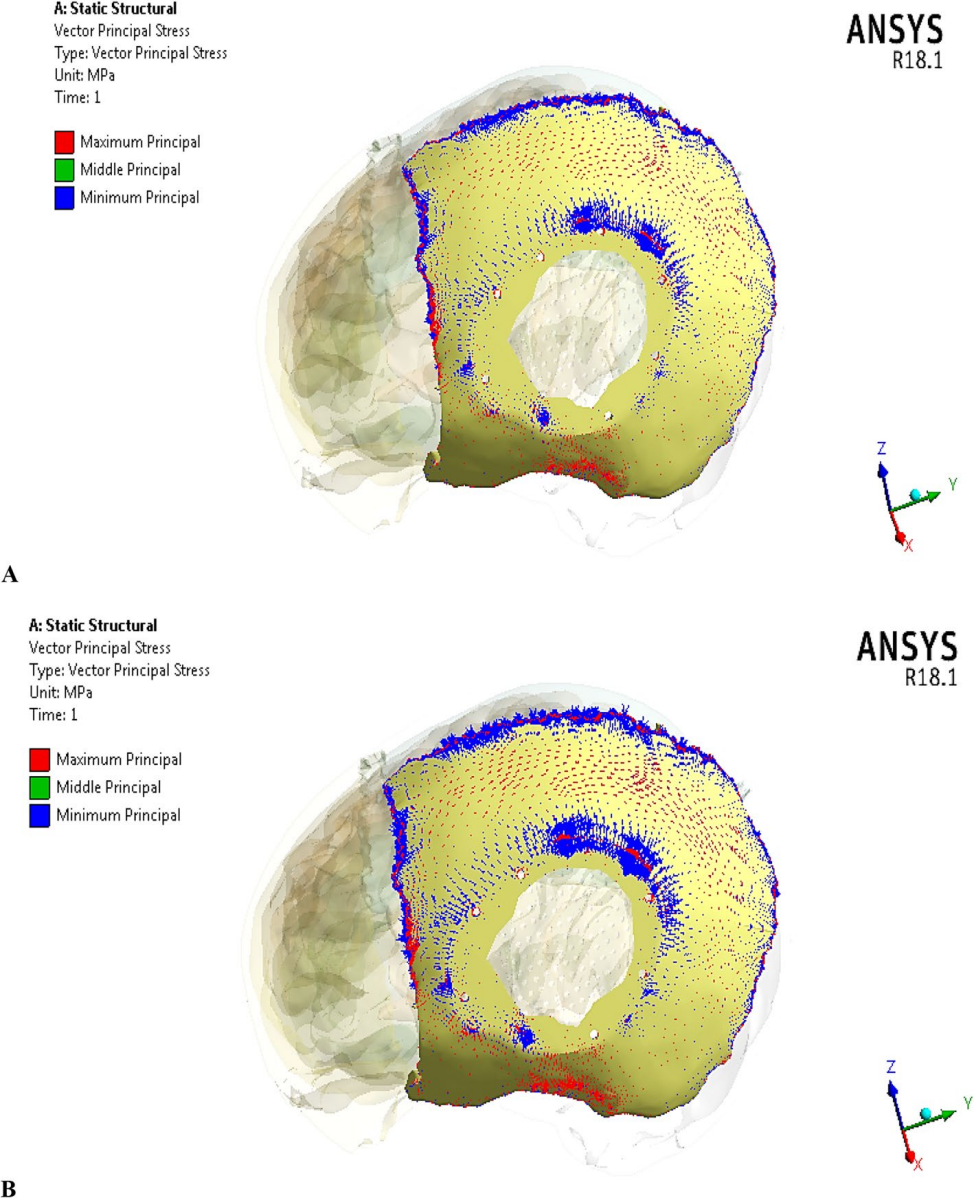
Principal stress vectors for left parietal bone using mesh implants: **A** CFR-PEEK 60%, and **B** PEEK. Where red arrows (tension) and blue arrows (compression)

Figure 13 illustrates the principal stress vectors for left parietal bone using plate CFR-PEEK 60% and PEEK implants. The red arrows indicated to the region with tension, while the blue arrows indicated to the region with compression. As illustrated in Fig. 13, the tensile stresses were more concentrated in the left and bottom regions of the left parietal bone. In the upper region, the tensile and compressive stresses were distributed in the sagittal suture. In the reconstructed portion, the compressive stresses were more distributed around the fixation points and the contact areas with the implant. It also observed a few concentrations of tensile stresses on the peripheral of the defective portion. By using the plate PEEK implant, the tensile and compressive stresses were more concentrated on the left parietal bone than by using the CFR-PEEK 60% implant. Figure 14 illustrates the principal stress vectors for left parietal bone using mesh CFR-PEEK 60% and PEEK implants. As shown in the Fig. 14, the compressive stresses were dominated and concentrated around the sutures, fixation points, and the contact areas with implants, while the tensile stresses were more concentrated in the bottom region of bone. In addition, the mesh PEEK implant concentrated the compressive stress more than the CFR-PEEK 60% implant.

### Stresses and strains on brain

**Fig. 15:**
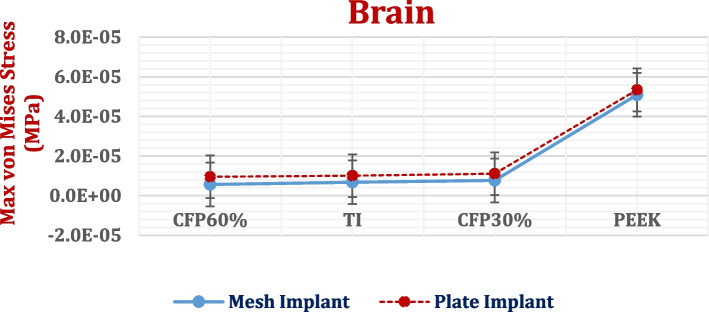
Max von Mises stresses (MPa) on the left hemisphere of brain using different implant designs and materials. (*P*-value < 0.05)

**Fig. 16:**
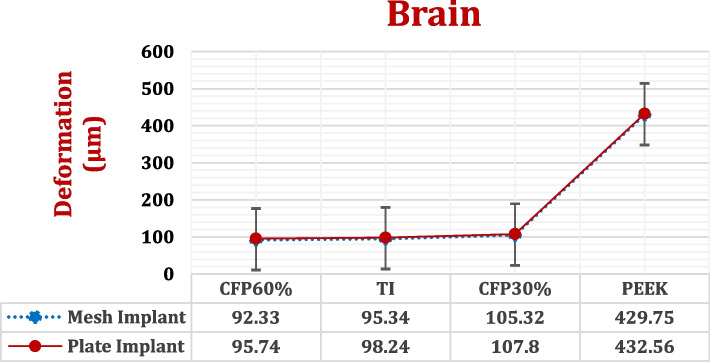
Total deformation (μm) of the left hemisphere of brain using different implant designs and materials. (*P*-value < 0.05)

**Fig. 17:**
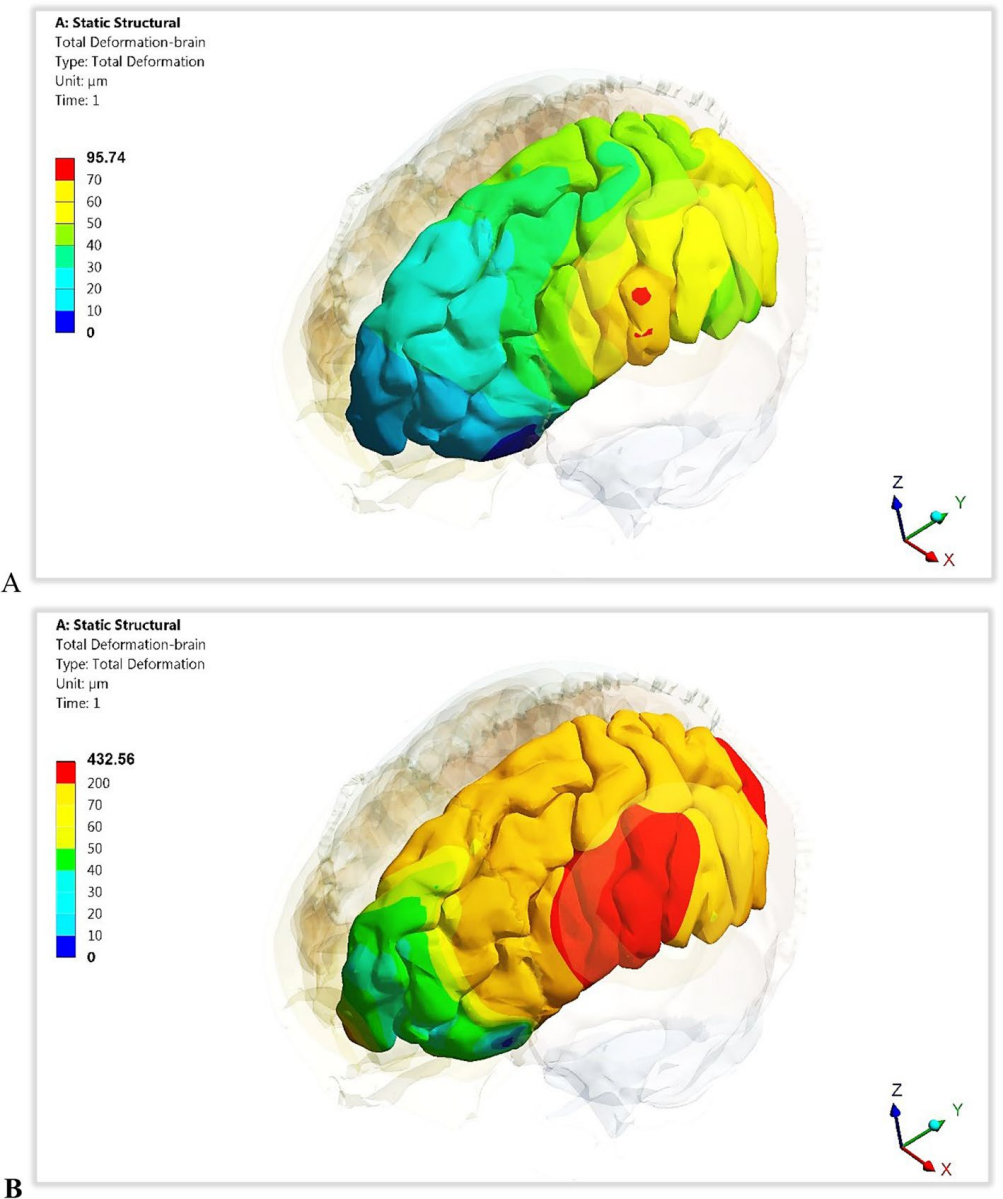
The distributions of total deformation (μm) of the left hemisphere of brain using plate implants: **A** CFR-PEEK 60%, and **B**) PEEK

This part aimed to calculate the maximum von Mises stresses, deformations, and maximum tensile and compressive stresses and strains on the left hemisphere of the brain (Figs. 15 and 16 and Tables 6 and 7), besides calculating the *p*-values. It is crucial to understand the alterations that occur in the brain due to the use of implants made of various designs and materials. Figure 17 illustrates the distributions of total deformations in the brain using plate CFR-PEEK 60% and PEEK implants. As shown in Figs. 16 and 17, the soft PEEK implants produced great deformations on the brain, compared to the stiff titanium and CFR-PEEK 60% implants.

**Table 6:** Peak tensile and compressive stresses (MPa) on the left hemisphere of brain using different implant designs and materials

		***TI***	***CFR-PEEK60%***	***CFR-PEEK30%***	***PEEK***
**Mesh Implant**	***Tensile***	1.23e-5	1.12e-5	1.41e-5	5.37e-5
	***Compressive***	1.35e-5	1.24e-5	1.55e-5	6.20e-5
**Plate Implant**	***Tensile***	1.49e-5	1.37e-5	1.63e-5	7.35e-5
	***Compressive***	1.66e-5	1.52e-5	1.81e-5	8.05e-5
***P*-Value**			**0.00023**	**0.00059**	**0.0009**

**Table 7:** Peak tensile and compressive strains (με) on the left hemisphere of brain using different implant designs and materials

		***TI***	***CFR-PEEK60%***	***CFR-PEEK30%***	***PEEK***
**Mesh Implant**	***Tensile***	3816	3478	4147	13,500
	***Compressive***	4059	3827	4229	15,110
**Plate Implant**	***Tensile***	5054	4564	5591	20,400
	***Compressive***	7130	6642	7621	26,278
***P*-Value**			**0.004**	**0.009**	**0.003**

In comparison to mesh titanium implant, mesh CFR-PEEK 60% implant reduced the maximum von Mises stress by 16.84%, and the total deformation by 3.15%. Moreover, mesh CFR-PEEK 60% implant reduced the peak tensile and compressive stresses by nearly 9%, and the peak tensile and compressive strains by 8.86% and 5.72%, respectively. By using plate CFR-PEEK 60% implant, the maximum von Mises stress and total deformation reduced by 5.54% and 2.54%, respectively, compared to those with the use of a plate titanium implant. In addition, the peak tensile and compressive stresses reduced by 8.05% and 8.43%, respectively, and the peak tensile and compressive strains reduced by 9.7% and 6.84%. The calculated *p*-value was lower than 0.05.

Compared to mesh titanium implant, mesh CFR-PEEK 30% implant increased the maximum von Mises stress by 13.74%, and the total deformation by 10.4%. Additionally, mesh CFR-PEEK 30% implant increased the peak tensile and compressive stresses by nearly 15%, and the peak tensile and compressive strains by 8.67% and 4.19%, respectively. By using plate CFR-PEEK 30% implant, the maximum von Mises stress and total deformation increased by 9.9% and 9.73%, respectively, compared to those with the use of a plate titanium implant. In addition, the peak tensile and compressive stresses increased by nearly 9.5%, and the peak tensile and compressive strains increased by 10.63% and 6.89%, respectively. The resultant *p*-value was lower than 0.05.

The brain was significantly impacted by PEEK implants. Regardless of its design, PEEK implant caused great increases in the stresses and strains on the brain. With the use of mesh PEEK implants, the tensile and compressive stresses were 5.37e-5 MPa and 6.2e-5 MPa, respectively, and the tensile and compressive strains were 13,500 με and 15,110 με, respectively. With the use of a plate PEEK implant, the tensile and compressive stresses were 7.35e-5 MPa and 8.05e-5 MPa, respectively, and the tensile and compressive strains were 20,400 με and 26,278 με, respectively. The resultant *p*-value was lower than 0.05.

## Discussion

The finite element method (FEM) has received considerable acceptance among medical practitioners and researchers as a technique for tackling biomechanical concerns [7]. FEM has emerged as a useful tool in the field of bone reconstruction construction for evaluating different designs and materials and optimizing the models [8]. Some authors [51, 52] have been concerned with using both finite element analysis (FEA), artificial neural networks (ANN) and Genetic algorithms (GA) to assist design optimization and enhance the reconstruction process.

The goal of Dhason et al*.* study [51] was to optimize the design of composite bone plates with the help of surrogate models to achieve selective stress shielding. Artificial neural network (ANN) surrogate models for bone displacements in all directions were developed using the simulated data from the finite element analysis. These models serve as the goal functions for multi-objective genetic algorithm-driven optimization. In Manickam et al*.* study [52], an S-type dynamic cage was designed with a different geometry of bone graft. The objective of this study was to reduce the stresses in the dynamic cage, so the risk of subsidence was controlled and optimized for the best suitable shape. A three-dimensional finite element model from C3-C6 was developed, and the S-type dynamic cage with the bone graft was virtually placed in the C4–C5 level. The stress level was optimized and the appropriate material and shape were assigned for each patient's cage and bone graft using the genetic algorithm.

For skull, anomalies or defects can be reshaped or corrected using a neurosurgical procedure known as cranioplasty [1]. Cranioplasty is often recommended for the treatment of posttraumatic injury or birth defects [2]. Cranioplasty can produce an intracranial stable state and stabilize intracranial pressure in addition to restoring the continuity, integrity, and previous appearance of the skull. Additionally, it lessens the negative effects of the defect and restores cranial nerve function, brain metabolism, and the protective layer surrounding the brain [3].

In the reconstruction process, a cranial implant with a specific design and material is placed and fixed with fixation points to restore the defective portion, stabilize intracranial pressure, and protect the brain. During the surgical procedures of implant placements (e.g., drilling, fitting), heat is produced which may influence the material characteristics, osseointegration, or efficacy of the implants. In addition, uncontrolled drilling may result in implant fracture and crippling from the fixation site. Hence, before surgery, many factors must be determined such as the velocity, pressure, diameter, and material of the driller, and the used coolant system to minimize the generated heat and avoid its related issues [53, 54].

Four common materials can be used in cranioplasty which are allografts or autografts, metal, polymers, and ceramic [6–8, 55]. Furthermore, two materials have emerged as the most used ones: titanium and polymethyl methacrylate (PMMA). Currently, titanium alloy (Ti-6Al-4 V) is the most often utilized repair material in the creation of cranial implants. It is well known that titanium alloys have excellent strength, corrosion resistance, durability, and biocompatibility. In the research of Valencia et al. [7], the optimization of cranial titanium implants was presented. Finite element analysis (FEA) was used to simulate sixty distinct models using the mechanical characteristics of an implant material made of grade 5 titanium alloy (Ti6Al4V). Twelve fixation points were included in the boundary conditions, and the material was exposed to intracranial pressure (ICP) conditions with a typical range of 10 mmHg. The designs were connected using an artificial neural network (ANN) to determine the maximum deformations. A generalized reduced gradient that minimized the amount of material was used to create optimal designs.

A case study is presented in Ameen et al*.* research [56] where a custom-made titanium cranial implant was designed using finite element method (FEM) and then manufactured using electron beam melting additive manufacturing. The results illustrated the successful manufacturing of thin custom cranial implants for skull reconstruction via electron beam melting technology. The manufactured implant had sufficient strength and weight while maintaining a good fit and aesthetics.

The potential for titanium to cause allergies is, however, one of its main drawbacks [19, 57]. Additional drawbacks include casting problems, surface modification requirements, aesthetic impairment, and incompatibility with imaging techniques. Therefore, alternative materials to titanium have been sought for use in the manufacturing of cranial implants such as polymers. Polymeric materials have been used in numerous medical applications as biocomposite matrices or soft tissue replacements. In neurosurgery, porous ethylene, and polymethyl methacrylate are often used polymers [22, 26, 58].

PMMA has been employed in surgical procedures because of its mechanical properties, cost, transparency, and ease of preparation. PMMA is used as a cement or pre-shaped solid implant in neurosurgery for treatments like cranioplasty and spinal stabilization because of its biocompatibility [59]. In Moncayo-Matute et al*.* research [60], new developments in terms of additive manufacturing, computational tools, and mathematical simulation techniques have been used for the restoration of a defective skull caused by an accident with a firearm using a custom-made PMMA implant. After medical follow-up for a considerable time after surgery, there were no complications. However, according to Zafar et al*.* research [59], PMMA is more prone to failure under high or sudden force due to its low modulus of elasticity and yield strengths compared to metallic and ceramic materials.

Currently, polyetheretherketone (PEEK) is a safe and scientifically certified material that can be used in bone reconstruction due to its good properties. In Rodríguez et al*.* research [61], finite element analysis of custom-made cranial implants was carried out under different design parameters and using PEEK and PMMA materials. The null hypothesis of this research demonstrated that both biomaterials would exhibit the minimum mechanical properties required to sustain direct impact trauma at the implant center, hence preventing critical deformations greater than 2 mm.

In Wan et al. research [62], 3D finite element models of defective skulls with four implant models were built. The four implant models were conventional titanium mesh, conventional wedge-shaped PEEK mesh, triangular parabolic titanium mesh with a hole button, and half wedge-shaped triangular parabolic PEEK mesh with a hole button. All the models were simplified and subjected to finite element analysis. Stress‒strain analysis was carried out to select a suitable cranial implant for cranioplasty. The results illustrated that under 500 N, the four implants and defective skulls did not experience breakdown or deformation.

In Bogu et al. research [30], a finite element study was conducted to investigate the effect of intracranial pressure on the implant under various situations and fixed at various points to achieve stability concerning the skull. The implant was stimulated with different materials (PEEK, PMMA, and Ti6Al4V), and the deformation and equivalent stress were extracted concerning fixation points. Twelve fine mesh models were simulated and analyzed in this research. From the results, it was observed that Ti6Al4V material showed low deformation while PEEK material showed less equivalent stress. However, both titanium alloy (Ti6Al4V) and Polyether-ether ketone(PEEK) implants showed good results.

In Santos et al*.* research [34], a cranial implant was designed to fulfil a defect created on the skull and then investigate its mechanical performance by integrating it into a 3D human skull model. The material chosen for the implant was the thermoplastic polymer (PEEK) which has been recently used in cranioplasty. Two criteria were analyzed using a numerical head model and an implant: the number of fixation screws that improve performance and guarantee the structural integrity of the implant, and the implant's ability to protect the brain in comparison to the integrated skull. The findings showed that, when taking into account the von Mises stress field and the displacement field on the interface between the implant and the skull, the model with eight screws performed better than any other screw configuration that had been examined. Jindal et al*.* [46] used the finite element method to compare several cranial implant materials (autologous bone, PMMA, PEEK, and Ti-6Al-4 V) fitted to a defective skull. The results show that PEEK and PMMA implants perform better than autologous bone implants and might be favoured over titanium implants.

The stiffness of PEEK materials can be improved by adding carbon fibres (carbon fiber reinforced PEEK (CFR-PEEK)) at certain percentages. CFR-PEEK composites combine the high strength of metals with the extensive biocompatibility and imaging compatibility of the polymers. Consequently, these composites have recently been used in many medical fields as substitutes for metallic restorations. These composites can be fabricated in several designs with various physical, mechanical, and surface properties [22, 24, 25]. In cranioplasty, one advantage of PEEK material and its composites is that the design of implants can be modified to create material properties similar to the skull, making these implants more appropriate than titanium implants [11, 30].

The choice of implant design used in cranioplasty is crucial factor [7, 8]. Mesh designs may offer more flexibility and better adaptability to irregular shapes, while plate designs could provide stronger support [7]. Additionally, mesh implants are lighter in weight compared to plate implants [8]. Some researchers recommended the use of mesh implants [7, 34], while others suggested plate implants [30, 43, 46].

The current research aimed to improve the process of skull reconstruction (cranioplasty) by using cranial implants from PEEK and carbon fiber reinforced PEEK 30 and 60%, in two designs, as alternatives to traditional titanium implants and evaluate their mechanical performance under an impact loading scenario, using the finite element method (FEM). Firstly, in the current paper, the intact model was validated with the model constructed by Pajic et al*.* [40], under a force of 7.7 KN which was applied perpendicularly to the forehead to stimulate a frontal hit. The maximum tensile and compressive stresses were extracted and compared with the limits of 92 MPa in tension and 133 MPa in compression, to estimate the risk of skull fracture. Compared to Pajic et al*.* [40], the extracted maximum von Mises stress, and maximum tensile and compressive stresses had lower values; However, the differences between the results did not exceed 10%. Another validation study was carried out with Godinho et al. study [50], using the same stimulated conditions and materials. A compressive vertical force of 750 N was applied to the frontal bone and the maximum and minimum principal strains were calculated. The result illustrated that the tensile and compressive strains on the loaded area had approximate ranges of 210–1439 με and 510 – 2242 με respectively. These values were within the ranges determined by Godinho et al. [50] which were 300-1600με and 600-2400με for tensile and compressive stresses respectively.

Secondly, after cranioplasty process, stress–strain analyses were conducted on the defective skull rehabilitated with a cranial implant with two designs (plate and mesh) and different materials which were the traditional titanium, soft PEEK, stiff PEEK composite (CFR-PEEK 60%), and semi-stiff PEEK composite (CFR-PEEK 30%). For evaluation, the total deformation, the maximum von Mises stresses and the peak tensile and compressive stresses and strains were calculated for the implant, brain and skull and compared to the allowable limits. The *p*-values were extracted to check the statistically significant of results.

From the extraction and evaluation of the results, the cranial implant's material and design greatly affected the stresses and strains generated on the skull and brain. Compared with plate implants, mesh implants increased the stresses on the implants (*P*-value < 0.05) and skull due to the presence of holes; however, they decreased the stresses on underlying brain tissues. By employing PEEK and CFR-PEEK 30% implants instead of titanium implants, the maximum stresses on the implants decreased as their elastic moduli (stiffness) were lower than titanium. Consequently, the maximum stresses and strains and the total deformations of the defective skulls and brains increased. By using stiff CFR-PEEK 60%, the maximum stresses on the implants increased; hence, the maximum stresses and strains of the skull and brain decreased. CF-PEEK 60% implants decreased also the total deformations of the skull and brain and therefore reduced the changes that occurred in their sizes and shapes compared to other materials. The *p*-values were lower than 0.05 which was considered statistically significant.

In the failure study of implants, the peak tensile and compressive stresses were calculated (in Table 8) and compared with the tensile and compressive yield limits (Table 1) under 2000 N force. The results indicated that titanium, CFR-PEEK 30 & 60% implants (whether mesh or flat) were not prone to fracture as the tensile and compressive stresses did not exceed the critical limits under the assumed loading scenario. For PEEK, mesh design was more susceptible to damage as the tensile and compressive stresses exceeded critical limits of 120 MPa under an impact force of 2000 N.

**Table 8:** Peak tensile and compressive stresses (MPa) on mesh and plate implants with different materials

		***TI***	***CFR-PEEK60%***	***CFR-PEEK30%***	***PEEK***
**Mesh Implant**	***Tensile***	157.93	159.32	135.55	121.22
	***Compressive***	225.79	230.31	197.54	122.64
**Plate Implant**	***Tensile***	53.43	55.55	34.87	29.47
	***Compressive***	77.67	79.25	69.97	60.86
***p*- Value**			**0.02**	**0.01**	**0.04**

For the skull, the peak tensile and compressive stresses were computed (Table 5) and compared to the tensile and compressive yield limits illustrated in Table 1. Furthermore, the peak tensile and compressive strains were compared with the physiological limits, as excessive strains can change the microstructure of bone and destroy the interfaces between implants and bone. A microstrain level exceeding 2500–3000 was typically indexed as the failure threshold for skull bone in tension and 4000–5000 in compression [49]. Compared to titanium implants, CFR-PEEK 60% decreased the stress and strain values on the skull, and the CFR-PEEK 30% slightly increased the stress and strain values but still within the acceptable limits. By using PEEK implants, the stresses and strains on the skull greatly increased. As illustrated in Table 6, the level of microstrain using mesh and plate PEEK implants exceeded the critical limits.

**Fig. 18:**
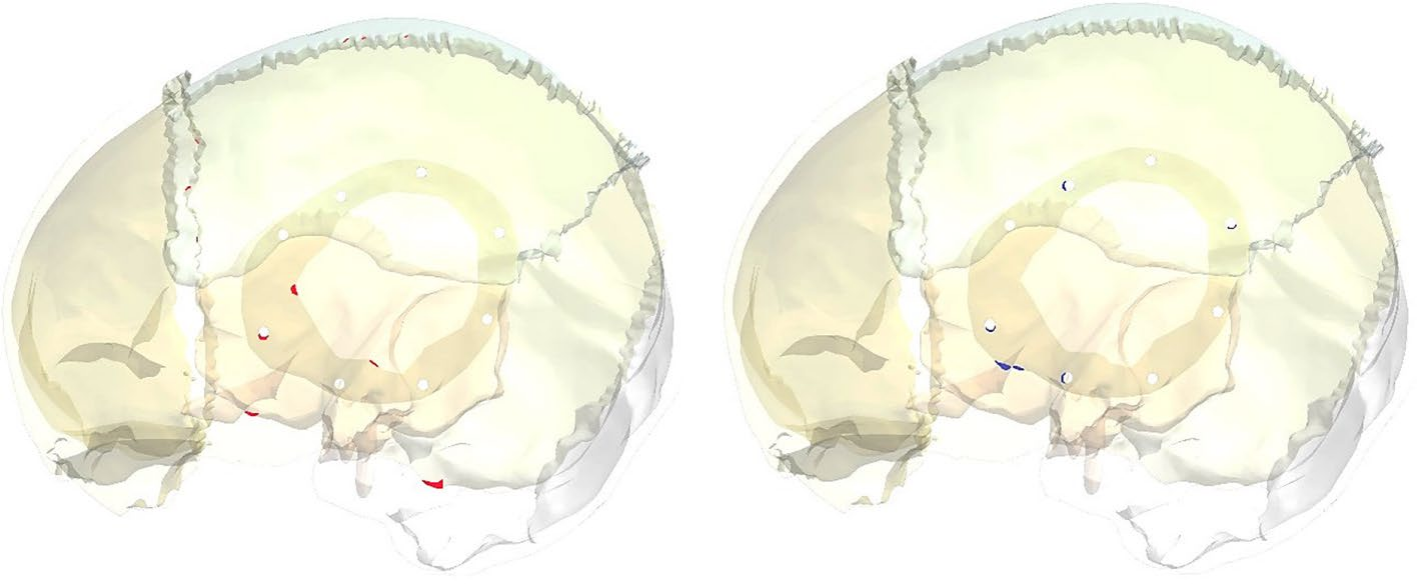
Expected fracture zones in the left parietal bone of skull due to excessive tensile strain (red) and compressive strain (blue) using plate PEEK implants

**Fig. 19:**
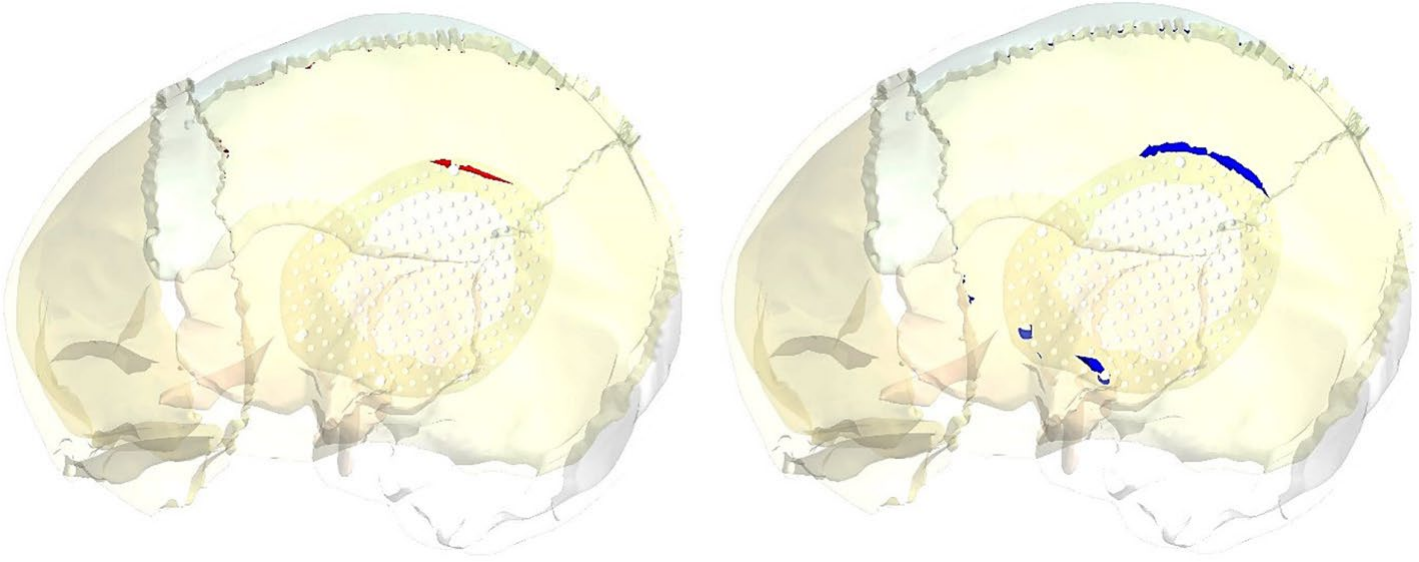
Expected fracture zones in the left parietal bone of skull due to excessive tensile strain (red) and compressive strain (blue) using a mesh PEEK implant

Figures 18 and 19 illustrated the expected fracture zones in the left partial bone of skull due to excessive tensile and compressive strains that exceeded the allowable limits, using plate and mesh PEEK implants, when an impact load of 2000N was applied perpendicular to implants. Fractures at the fixation points were expected while using the plate PEEK implant. By using mesh PEEK implant, the fractures were expected around the fixation points, and in the contact area with the left parietal bone.

For the brain, by using all materials and designs, the peak tensile and compressive stresses were within the allowable limits of approximately 1 kPa in tension and 50 kPa in compression, respectively [41, 42]. Furthermore, the tensile and compressive strains did not exceed the microstrain limits. These limits were 100,000, 200,000 and 250,000, corresponding to reversible damage, functional damage, and structural damage, respectively, under both tension and compression [34].

This study had limitations such as full osseointegration was assumed between the implant and the surrounding bone, which might not be feasible in real situations. Additional limitations were related to the layout and positioning of the implants since any modifications could change the results. Furthermore, the process used to determine the load and boundary conditions was theoretical and might not accurately reflect a clinical scenario in the real world. Other limitation was related to the lack of experimental validation.

Further experimental and surgical studies, besides dynamic finite element investigations, are required in the near future on other cases of skull defects, taking into consideration the patient's age, asymmetrical defects, and variations in bone density, as well as making follow-up reports after surgery.

## Conclusion

The purpose of this research was to conduct finite element investigation to enhance the skull reconstruction process by using PEEK and CFR-PEEK 30 and 60% in the production of cranial implants as alternatives to titanium, under impact loading scenario. Based on the results and within the limitations of the current study, the following conclusions were explored:

1. Compared with mesh implants, plate implants decreased the stresses on implants and skull; however, they increased the stresses on underlying brain tissues.
2. Titanium, CFR-PEEK 30 & 60% implants (whether mesh or flat) were not prone to fracture as the tensile and compressive stresses did not exceed the critical limits.
3. For PEEK, mesh design was susceptible to damage as the tensile and compressive stresses exceeded the critical limits of 120 MPa under an impact force of 2000 N.
4. The CFR-PEEK 60% implants produced the lowest values of tensile and compressive stresses and strains and the lowest deformations of the skull and brain.
5. The PEEK implants produced the highest values of tensile and compressive stresses and strains and the greatest deformations of the brain and skull.
6. The CFR-PEEK 30% implants slightly increased the stress and strain values on the skull and brain compared to titanium, but these values did not exceed the critical limits.
7. The distribution of stresses in skull and brain by using CFR-PEEK 30% and CFR-PEEK 60% implants were nearly similar to those using titanium implants.
8. The contact area between the PEEK implant and the left parietal bone of the skull was expected to be damaged, due to the excessively generated strains. Hence, plate and mesh PEEK implants were not recommended to be used in cranioplasty because they exerted high stresses and strains on the skull and brain.
9. By utilizing all implants, the tensile and compressive stresses and strains on the brain, were within the allowable limits.
10. In comparison to titanium implants, the calculated *p*-values by using CFR-PEEK 30& 60% and PEEK implants were lower than 0.05, referring to the statistical significance of the results.

Finally, it was concluded that in the skull reconstruction process, the PEEK composites (semi-stiff CFR-PEEK 30% implant and stiff CFR-PEEK 60% implant), especially when used with plate designs, were recommended as alternatives to titanium implants. These PEEK composites are known for their superior properties, biocompatibility, and lack of clinical problems. They do not induce inflammation, are safe for nearby structures, and are not prone to deformation or fracture from rapid impacts. Furthermore, these composites can be formed into a variety of shapes and sizes, and have a broad range of mechanical, surface, and physical properties. This research anticipates main benefits from using CFR-PEEK 30% and 60% implants, such as increased design freedom, reduced overall system cost, avoidance of clinical issues, easier follow-up after surgery, and enhanced performance.

## Data Availability

The data used to support the findings of this study are included within the article.
