# Correction: Cranial reconstruction utilizing polymeric implants in two different designs: finite element investigation

**DOI:** 10.1186/s12891-025-08334-3

**Published:** 2025-01-28

**Authors:** Yomna H. Shash

**Affiliations:** Biomedical Engineering Department, Faculty of Engineering, Helwan University, Cairo, Egypt

**Correction:** ***BMC Musculoskelet Disord*** **25, 935 (2024)**

10.1186/s12891-024-08066-w

Following publication of the original article [1], the author corrected the incorrect captions captured for Figs. 1, 2, 3, 7 and 8.

**The correct captions of Figs. 1**,** 2**,** 3**,** 7 and 8**:

**Fig. 1** Model parts: (A) brain, (B) defective skull, (C) mesh implant, (D) plate implant, (E) eight fixation mini-screws, (F) Final model with mesh implant, and (G) Final model with plate implant.

**Fig. 2** Bonded connection between left parietal bone and implant.

**Fig. 3** Mesh setting.

**Fig. 7 M**aximum von Mises stresses (MPa) on mesh and plate implants with different materials. (P-Value < 0.05)

**Fig. 8** The distributions of maximum and minimum principal stresses (MPa) on plate CFR-PEEK 60% implant.

**The incorrect captions of Figs. 1**,** 2**,** 3**,** 7 and 8**:

**Fig. 1** Illustration of the study period.

**Fig. 2** Flow chart of the patients included in the study. The patient exclusion was conducted if the patient died before the operation, or a patient undertook anesthesia other than general, thereby, not providing the LOS outcome variable.

**Fig. 3** Flowchart of detailing how the prediction model in the postoperative length of stay was built.

**Fig. 7** The SHAP dependence plots. The plot displays the variable that interacted with the target. The x-axis represents the feature value and the y-axis represents the SHAP value of the feature. The color corresponds to the value of the interacted feature. a The direct bilirubin blood laboratory test predominantly interacts with the total bilirubin blood laboratory test. b The department change mostly interacts with heparin sodium medication. c The calcium chloride medication primarily interacts with blood glucose stick laboratory tests. d Gender mainly interacts with department change. e The removal of other organ diagnoses mostly interacts with the direct bilirubin blood laboratory test. f The citric acid and magnesium carbonate medication principally interacts with the cardiothoracic surgery department.

**Fig. 8** SHAP force plots. a A patient in the first row provides a prediction of 5.94. b A patient in row 201 produces a prediction of 10.79. c A patient in row 501 yields a prediction of 5.83. d A patient in row 5001 supplies a prediction of 4.10.

The original article [1] has been updated.
